# Epothilone B impairs functional recovery after spinal cord injury by increasing secretion of macrophage colony-stimulating factor

**DOI:** 10.1038/cddis.2017.542

**Published:** 2017-11-02

**Authors:** Liang Mao, Wei Gao, Shurui Chen, Ying Song, Changwei Song, Zipeng Zhou, Haosen Zhao, Kang Zhou, Wei Wang, Kunming Zhu, Chang Liu, Xifan Mei

**Affiliations:** 1 Department of Oncology, The First Affiliated Hospital of Jinzhou Medical University, Jinzhou 121000, People's Republic of China; 2 Key Laboratory of Medical Tissue Engineering of Liaoning Province, The First Affiliated Hospital of Jinzhou Medical University, Jinzhou 121000, People's Republic of China; 3 Department of Basic Medical Sciences, Jinzhou Medical University, Jinzhou 121000, People's Republic of China; 4 Jinzhou Medical University, Jinzhou 121000, People's Republic of China; 5 Department of Hand Surgery, The First Affiliated Hospital of Jinzhou Medical University, Jinzhou 121000, People's Republic of China; 6 Department of Orthopedic Surgery, The First Affiliated Hospital of Jinzhou Medical University, Jinzhou 121000, People's Republic of China; 7 Department of Endocrinology, The First Affiliated Hospital of Jinzhou Medical University, Jinzhou 121000, People's Republic of China

## Abstract

The microtubule-stabilizing drug epothilone B (epoB) has shown potential value in the treatment of spinal cord injury (SCI) through diverse mechanisms. However, it remains elusive why a limited overall effect was observed. We aim to investigate the limiting factors underlying functional recovery promoted by epoB. The same SCI model treated by epoB was established as discussed previously. We used a cerebrospinal fluid (CSF) sample to assess the changes in cytokines in milieu of the SCI lesion site after epoB treatment. We then analyzed the source of cytokines, the state of microglia/macrophages/monocytes (M/Ms), and the recruitment of neutrophil in the lesion site by using the results of antibody array. Following these findings, we further evaluated the motor functional recovery caused by the reshaped microenvironment. Systemic administration of epoB significantly increased levels of several cytokines in the CSF of the rat SCI model; macrophage colony-stimulating factor (M-CSF) secreted by intact central nervous system (CNS) cells was one of the cytokines with increased levels. Along with epoB and other cytokines, M-CSF reshapes the SCI milieu by activating the microglias, killing bone marrow-derived macrophages, polarizing the M/M to M1 phenotype, and activating downstream cytokines to exacerbate the SCI injury, but it also increases the expression of neurotrophic factors. Anti-inflammatory therapy using a neutralizing antibody mix shows encouraging results. Using *in vivo* experiments, our findings indicate that epoB inhibits the SCI functional recovery in many ways by reshaping the milieu, which counteracts the therapeutic efficacy that led to the limited overall effectiveness.

Epothilone B (epoB) is an ideal drug for the treatment of spinal cord injury (SCI), which can reduce scar formation in the lesion site, reactivate the axons’ regeneration potential, and is convenient for operation.^1^ In theory, epoB has a therapeutic role in SCI by divergent mechanisms. First, epoB is a blood–brain barrier-permeable microtubule-stabilizing drug that is absorbed in the central nervous system (CNS) after being administered.^1^ Second, through neuron-specific protein Tau expression, epoB promotes axon elongation and reduces fibrotic scarring simultaneously.^1^ Third, epoB reduces axonal dieback and promotes serotonergic axon regrowth. Thus, the rat SCI model exhibits functional recovery after epoB administration.^1^ It seems epoB matches every step of SCI treatment perfectly.^1^

However, the overall efficiency of epoB is not quite so satisfactory. As a matter of fact, the number of footfall errors was reduced ~50% on the horizontal ladder compared with the control. These effects could be counteracted by ablation of 5-hydroxytryptamine.^1^ Furthermore, epoB does not appear superior to any other monotherapies.^2, 3, 4^ To further evaluate the therapeutic limitation of epoB, we can refer to the similar microtubule-stabilizing drug taxol, which was tested as a treatment for SCI for many years.^5^ Hellal *et al.*^6^ showed that taxol also possessed multitargeted abilities in treatment of SCI similar to epoB. Nevertheless, recently an independent evaluation reported that taxol is able to reduce the scar formation in the SCI lesion site; however, it does not possess the ability to promote serotonergic axon growth and protect neurons from damage.^7^ It is still unclear why these contradictions occur.

A fundamental question is whether epoB is beneficial for the SCI functional recovery overall and it also consists of some detrimental factors. Here, we found the changes of cytokines in the cerebrospinal fluid (CSF) instead of lesion site milieu. We investigated the source and function of elevated macrophage colony-stimulating factor (M-CSF) focus on the microglia/macrophages/monocytes (M/Ms) in the SCI lesion site after epoB treatment. We further evaluated the downstream cytokine LIX (CXCL5) focused on the effects of neutrophil recruitment. These results indicate that combined with elevated cytokines, epoB suppresses SCI functional recovery by killing bone marrow-derived macrophages (BMDMs), activating microglia, polarizing M/M to M1 phenotype, and recruiting neutrophils to increased lesion inflammation burden. Unlike previous reports, these effects retarded the functional recovery after SCI.

## Results

### EpoB increases levels of several cytokines in CSF after SCI

**Figure 1:**
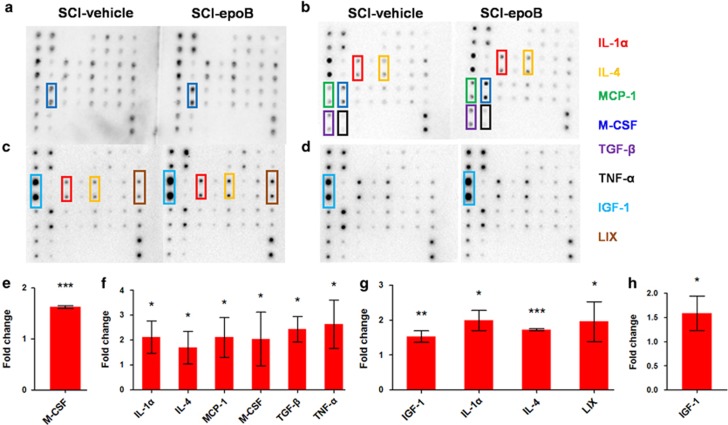
Eight cytokines increased in CSF of SCI models after epoB treatment. The doublet spots in the membranes indicate each cytokine. (**a**–**d**) The CSF of SCI-epoB or SCI-vehicle was detected continuously. (**a**) One day after epoB treatment, only elevated M-CSF was observed. (**b**) Three days after epoB administration, six cytokines were elevated at different levels, including IL-1*α*, IL-4, M-CSF, MCP-1, TNF-*α*, and TGF-*β*. (**c**) Over time, IGF-1 and LIX increased in CSF 7 DPE. (**d**) Two weeks after epoB treatment, only IGF-1 was upregulated. (**e–h**) Densitometric analysis of increased cytokines 1 (**e**), 3 (**f**), 7 (**g**), and 14 (**h**) DPE. *n*=3 per group; *P-value <0.05; **P-value <0.01; ***P-value <0.001 (one-way ANOVA)

Because biologically active cytokines of SCI milieu exist in the SCI lesion mainly, and the cytokines of SCI lesion differ very little with CSF, we assessed the profiles of expression of cytokines by cytokine protein array with CSF from the SCI model treated with epoB (*n*=3) or solvent control (*n*=3). Interestingly, epoB-treated SCI rats were only M-CSF elevated compared with the control at 1 day post epoB treatment (DPE) (Figures 1a and e); however, both anti- and proinflammatory molecules including interleukin-1*α* (IL-1*α*), IL-4, M-CSF, monocyte chemoattractant protein-1 (MCP-1), tumor necrosis factor-*α* (TNF-*α*), and transforming growth factor-*β* (TGF-*β*) were elevated in the CSF of epoB-treated SCI models at 3 DPE (Figures 1b and f). In addition to IL-1*α* and IL-4, insulin-like growth factor-1 (IGF-1) and LIX were elevated 7 days after epoB treatment (Figures 1c and g) and only IGF-1 increased 14 DPE in CSF (Figures 1d and h). According to the CSF cytokine profiling data, M-CSF is the earliest rise in CSF. Based on these results we have reason to assume that the elevated M-CSF may be the key point for interpreting the limited efficacy of epoB administration in SCI. To clarify the biological effects of elevated M-CSF in milieu, we need to identify the value, duration, and cell origin of elevated M-CSF at first.

### Elevated M-CSF derived from intact CNS astrocytes, T cells, M/Ms, and primarily ependymal cells lasting 4 days

**Figure 2:**
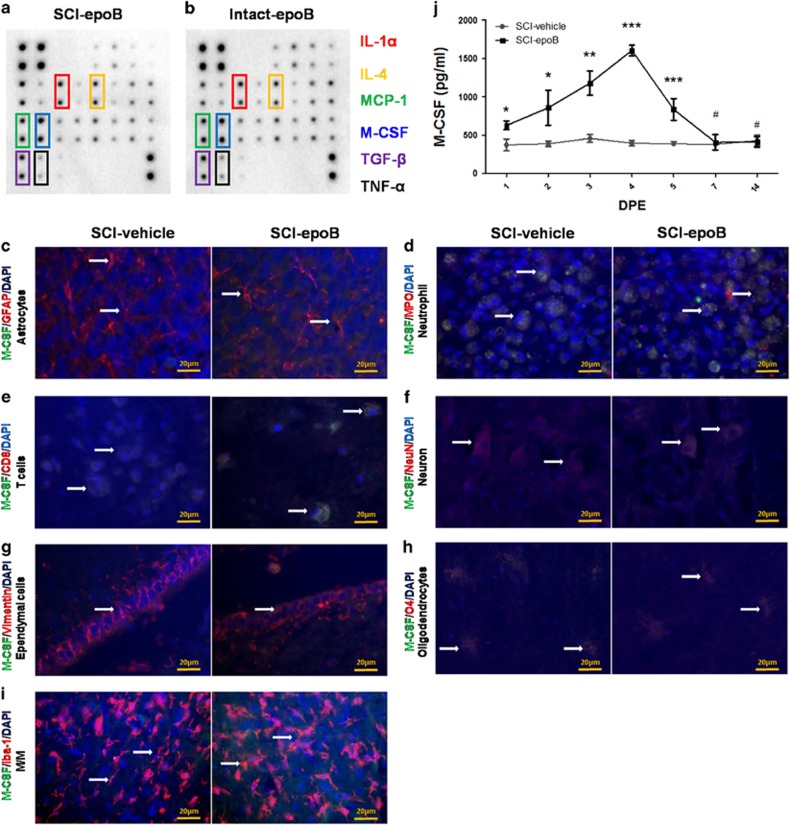
Intact CNS astrocytes, T cells, ependymal cells, and M/Ms mainly contribute to the increasing secretion of M-CSF. (**a** and **b**) Two membranes show that the CSF cytokine profile of SCI-epoB (**a**) or intact-epoB (**b**) were unchanged in six cytokines 3 DPE. (**c**–**i**) Representative fluorescent staining images show SCI-epoB and SCI-vehicle 3 DPE. Double staining of particular cell types of CNS and M-CSF plus DAPI labeling for nuclei shows astrocytes (**c**), T cells (**e**), neurons (**f**), ependymal cells (**g**) and M/Ms (**i**) increase the secreting of M-CSF. Others cells of neutrophils (**d**) and oligodendrocytes (**h**) exhibit no change on M-CSF secretion. Arrows indicate examples of double-labeled cells. (**j**) Quantitative analysis of M-CSF expression in the CSF by ELISA. Means±S.D.; *n*=3 per group in (**a**) and (**b**). *n*=6 per group in (**c**)**–**(**i**). *n*=3 per group in (**j**); *P-value <0.05; **P-value <0.01; ***P-value <0.001 (one-way ANOVA)

We next performed the CSF cytokine profile using the epoB-treated SCI model (*n*=3) or intact model (*n*=3). Interestingly, the elevated six cytokines 3 DPE of epoB-treated SCI model shows no obvious difference compared with the epoB-treated intact model (Figures 2a and b). These data suggest that the upregulated six cytokines of 3 DPE did not generate from the injured CNS. For further evaluating the cellular sources of elevated M-CSF, we conducted immunohistofluorescence double staining (*n*=6) using M-CSF and specific antigen of CNS cells. It is shown that astrocytes, T cells, M/Ms, ependymal cells, and neurons were the main sources of elevated M-CSF at 3 DPE (Figures 2c, e–g, and i). Meanwhile, enzyme-linked immunosorbent assay (ELISA) experiments reveal that levels of M-CSF rose 1 DPE and lasted up to 4 days at least (*n*=3); the concentration of M-CSF peaked at ~1.6 ng/ml at 4 DPE (Figure 2j).

### EpoB and elevated M-CSF exhibit cytotoxic effects rather than proliferation in SCI lesion M/Ms

**Figure 3:**
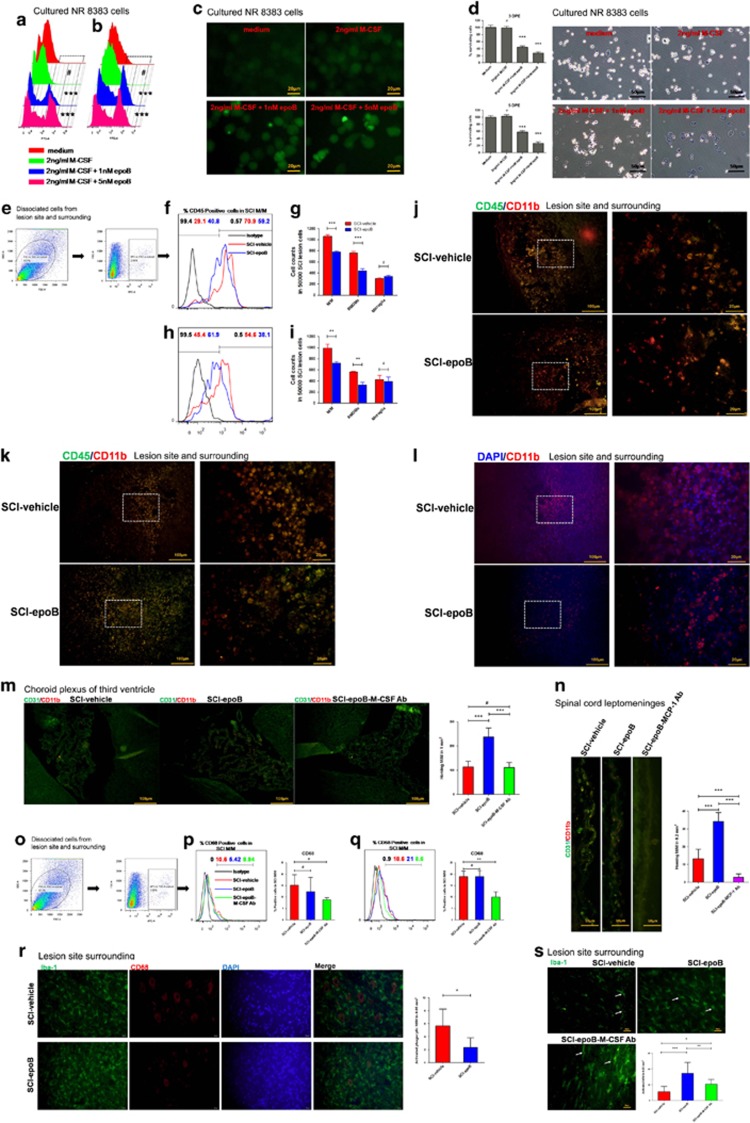
SCI milieu exhibits cytotoxic to homing macrophages rather than promotes proliferation and in contrast activates microglias in acute stages after epoB treatment. (**a**–**d**) NR8383 cell line stained by CFSE was treated by M-CSF plus epoB. Either 3 (**a**) or 5 (**b**) DPE cells were analyzed by FC (Pearson's *χ*^2^ test or Fisher’s exact test). (**c**) Representative images of treated NR8383 cells 3 DPE. (**d**) Treated NR8383 cells were measured by MTT and trypan blue 3 DPE, indicating that the elevated M-CSF were unable to stimulate M/M division; however, epoB exhibits cytotoxic effects to M/M (one-way ANOVA). (**e**–**l**) SCI milieu reduces BMDMs after epoB treatment. (**e**) Representative plots showing the separating process of M/M in the SCI lesion. Representative plots (**f**) and quantification (**g**) of BMDMs (CD11b^+^ CD45^+^) and microglia (CD11b^+^ CD45^−^) show ~40% reduction of BMDMs in the SCI lesion milieu 3 DPE. Panels h and i show this tendency decreases 7 DPE. (**j** and **k**) Representative immunofluorescence images of BMDMs and microglias in the SCI lesion site 3 (**j**) and 7 (**k**) DPE. (**l**) Representative immunofluorescence images of peripheral circulation-labeled M/M in SCI lesions 3 days after epoB treatment (one-way ANOVA). (**m** and **n**) Representative immunofluorescence images and quantification of elevated M-CSF and MCP-1 recruit M/M via brain-ventricular choroid plexus (**m**) and SC leptomeninges (**n**) (Mann–Whitney *U*-test). (**o**–**s**) Elevated M-CSF-activated resident microglias but not homing microphages. (**o**) Representative plots show the separating process of M/M in the SCI lesion. Representative plots and quantification of M/M phagocytic phenotypes (CD11b+ CD68+) analyzed by FC in SCI lesions 3 (P) or 7 (**q**) DPE. Representative immunofluorescence images of phagocytic phenotype M/M in the SCI-vehicle and SCI-epoB 3 DPE (**r**). Representative immunofluorescence images of activated phenotype M/M in the SCI-vehicle and SCI-epoB 3 (**s**) DPE (one-way ANOVA). Mean±S.D.; *n*=6 per group in (**e**)–(**k**). *n*=3 per group in (**l**). *n*=6 per group in (**m**–**s**); (**a**–**d**) were conducted by using cultured NR8383 cell line. (**e**–**i**) were conducted by using dissociated SCI lesion site and surrounding cells (0.5 cm length SC using the lesion site as a center). (**j**–**l**) represent SCI lesion site and surrounding (using the lesion site as a center). (**m**) and (**n**) represent choroid plexus of third ventricle and SC leptomeninges, respectively. (**o**–**q**) were conducted by using dissociated SCI lesion site and surrounding cells (0.5 cm length SC using the lesion site as a center). (**r**) and (**s**) analyzed in SCI lesion surrounding of epicenter±1000 *μ*M. *P-value <0.05; **P-value <0.01; ***P-value <0.001

M-CSF is responsible for the survival, differentiation, activation, proliferation of M/Ms, and recruitment of M/Ms, mainly to lesions.^8^ Accordingly, we next evaluated the biological effects of elevated M-CSF on the above aspects caused by epoB in SCI. For some experiments requiring cultured purified M/Ms, we used the rat alveolar macrophage cell line NR8383 to evaluate the proliferation of M/Ms. First, we determined if elevated M-CSF caused expansion in the M/Ms. NR8383 cells were stained by carboxyfluorescein diacetate succinimidyl ester (CFSE) followed by 2 ng/ml M-CSF plus PBS and 1 nM, or 5 nM epoB treatment *in vitro*. Either 3 or 5 days after stimulating, the NR8383 cells were detected by flow cytometry (FC) for cell proliferation. As a matter of fact, even at the highest level and longest stimulation used, M-CSF did not significantly stimulate M/M division compared with controls; on the contrary, in epoB-treated NR8383 cells, division reduced (Figures 3a–c). To clarify the reason for reduction in growth rate, we further performed the cell viability experiment *in vitro*. As Figure 3d shows, more than half of NR8383 cells were killed by 1 or 5 nM epoB.

Following this route, we further assessed the cytotoxic effect of epoB in SCI lesion M/M *in vivo*. We separated SCI lesion M/M using CD11b antibody by FC (*n*=6) (Figure 3e). Next, we distinguished BMDMs from resident microglias using CD45 antibody. As expected, systemic administration of epoB reduced SCI lesion BMDMs (Figures 3f and h). Unexpectedly, this effect was not found in resident microglias (Figures 3g and i). Immunofluorescence staining (*n*=6) further shows a decrease in BMDMs but not microglias at 3 and 7 DPE (Figures 3j and k). Furthermore, this effect in BMDMs was reinforced by APC-labeled peripheral monocytes homing to the SCI lesion (*n*=3) (Figure 3l). The next fundamental question is whether the SCI milieu is cytotoxic to BMDMs after epoB treatment or whether epoB kills peripheral monocytes, thereby resulting in reduced recruitment of M/M into the SCI lesion.

### The elevated M-CSF and MCP-1 promote recruitment of M/M into the SCI lesion

M-CSF and MCP-1 recruit two different types of M/M that go through two different paths to the SCI site.^9^ MCP-1, also known as CCL2, is a chemotactic factor that attracts M1 to the SCI lesion site through spinal cord (SC) leptomeninges.^9^ The corresponding cytokine is M-CSF, which recruits M2 to the SCI lesion site through choroid plexus. In our research, both M1 and M2 chemotactic factors elevated at 3 DPE. We therefore tested whether the elevated two factors promote the homing process of M/M to the SCI lesion. Immunofluorescent staining of the third ventricle choroid plexus (*n*=6) shows an increase of homing M/M in SCI-epoB compared with SCI-vehicle. On the contrary, these recruitment effects could be reduced by M-CSF-neutralizing antibody treatment (Figure 3m). Similar to M-CSF, Figure 3n shows that M/Ms are enriched by SC leptomeninges (*n*=6) of the SCI-epoB group; these effects could be weakened by corresponding neutralizing antibody treatment. Quantification analysis shows that the elevated two factors M-CSF and MCP-1 contribute to ~2- and 2.5-fold increase in homing M/M, respectively. These findings suggested that both increased M1 and M2 cells infiltrated in the SCI microenvironment after epoB administration, and the homing M/Ms were killed by epoB mainly in the CNS.

### EpoB plus elevated M-CSF activate microglias and suppress microglia phagocytosis in CNS

To investigate the active effect promoted by elevated M-CSF in M/Ms, we used the CD11b, CD68, and Iba-1 staining to identify the activated state. CD11b is a pan-marker of M/Ms, CD68 is a macrophage marker related to its phagocytic function, and Iba-1 represents the M/M activation. Meanwhile, they are all affected by total amount of M/Ms. As a matter of fact, FC (*n*=6) shows that phagocytic markers of M/M CD68 in SCI lesions were not changed significantly at 3 or 7 DPE between the SCI-epoB- and vehicle-treated groups (Figures 3p and 3q). However, phagocytic markers could be reduced by the administration of M-CSF-neutralizing antibody. The above results led us to consider morphological changes in M/Ms; we next focused on the immunostaining (*n*=6) for CD68 and Iba-1. Interestingly, in the SCI-vehicle group, CNS M/M phagocytic phenotypes were altered to turgid and smooth. By contrast, phagocytic M/M experience shrinkage and size reduction in the SCI-epoB group (Figure 3r). Furthermore, SCI-vehicle-group M/M tend to be in a resting state mainly; on the contrary, M/M in the SCI-epoB group become active. Typical representation of the resting state of M/M is slender and thin; nevertheless, the M/M active state is rounded and the tail is spread, which could be transformed by M-CSF-neutralizing antibody partly (*n*=6) (Figure 3s). These results reveal that the elevated M-CSF plus epoB changed the active and phagocytic function via altering cell morphology rather than changing its number. By reason, BMDMs’ phagocytic and active states could not be distinguished by cellular morphology.^10^ We have reason to believe that changes in phagocytic and active morphology occur mainly in microglias.

### The remodeled microenvironment polarized homing and resident M/M to M1 phenotype

**Figure 4:**
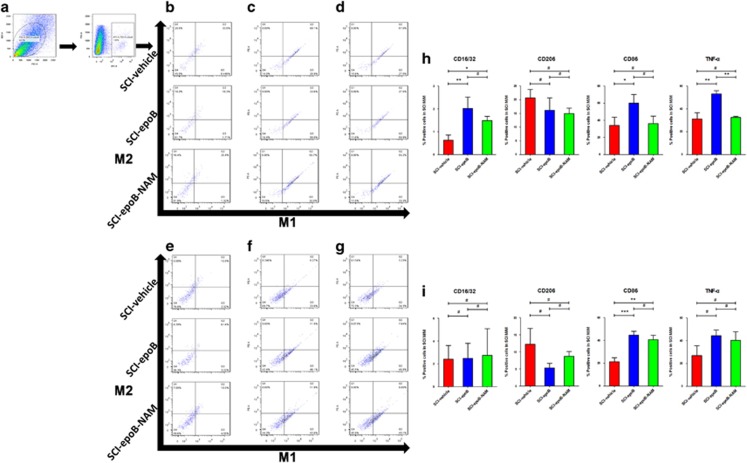
SCI lesions polarize M/M from M2 to M1 phenotype 3 days after epoB treatment; the neutralizing antibody mix (NAM) partly reverses this polarization. (**a**) Representative plots showing the separating process of M/M in SCI lesions. Representative plots of M1 and M2 polarization are distinguished by (**b**) CD16/32 plus CD206, (**c**) CD86 plus CD163, and (**d**) TNF-*α* plus TGF-*β* in the SCI-vehicle, SCI-epoB, and SCI-epoB-NAM lesion sites 3 DPE. This experiment was conducted on the above group with the markers of (**e**) CD16/32 plus CD206, (**f**) CD86 plus CD163, and (**g**) TNF-*α* plus TGF-*β* 7 DPE. (**h–i**) Quantification of M/M polarization analyzed by CD16/32, CD86, CD163, and TNF-*α* at 3 DPE (**h**) and 7 DPE (**i**). Means±S.D.; *n*=6 per group; *P-value <0.05; **P-value <0.01; ***P-value <0.001 (one-way ANOVA)

The previous results raised another interesting question: the infiltrating and resident M/Ms are exposed to the anti-inflammatory as well as the proinflammatory milieu simultaneously, so how does the remodeled milieu re-educate M/M phenotype? Is the re-educated M/Ms detrimental or beneficial for SCI recovery? These questions led us to detect a series of M1 and M2 phenotypes in homing and resident M/Ms in SCI lesions 3 or 7 DPE. Based on previous literature, we chose CD16/32, CD86, and TNF-*α* as M1 markers and CD206, CD163, and TGF-*β* as M2 markers.^11^ Cells isolated from SCI lesions were triple-labeled M/M pan-marker CD11b and M/M phenotype markers for FC analysis (*n*=6). Figures 4b–d showed that CD16/32, CD86, and TNF-*α* expressed an M/M increase and was associated with decreasing M/M with CD206 expression 3 DPE. However, CD163- and TGF-*β*-expressed M/M was not detected. It is worth noting that using all six neutralizing antibodies, M/Ms tend to possess less M1 phenotype compared with SCI-epoB group 3 DPE. The findings indicate that beyond our antibody-array-detected cytokines, the rest of the cytokines tend to be in the anti-inflammatory state. The polarization is weakened; meanwhile, the neutralizing antibody mix tends to be unable to counteract the inflammatory milieu 7 DPE (Figures 4e–g).

### Elevated LIX mediates neutrophil recruitment and aggravates SCI

**Figure 5:**
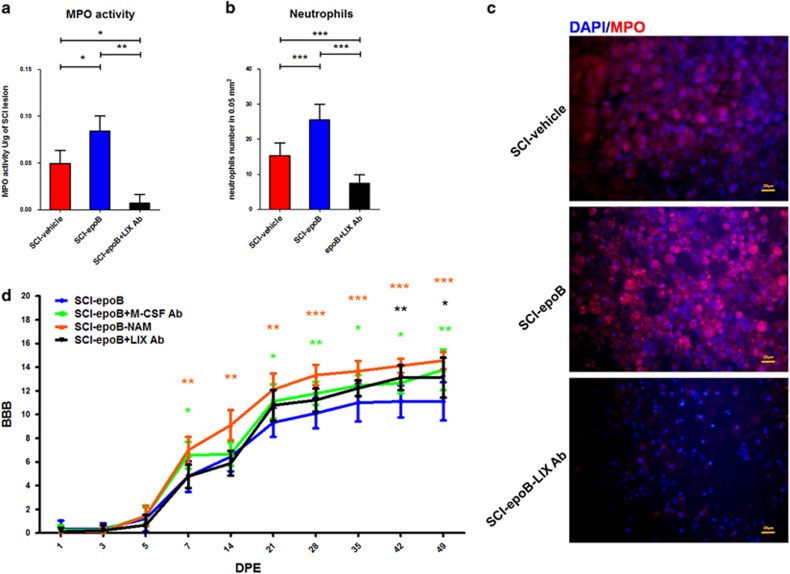
LIX increases the burden of inflammation by recruiting neutrophils to the lesion site and locomotion assessment. (**a**) Graphs show the myeloperoxidase activity of lesion sites of SCI-epoB, SCI-epoB treated by IL-1*α* plus TNF-*α* antibody, and SCI-epoB treated by LIX antibody. (**b**) Quantification and (**c**) representative images of neutrophil recruitment in the lesion sites of three groups. (**d**) Motor function assessed by BBB continuously. Data were expressed as mean±S.D.; *n*=6 per group in (**a**)**–**(**c**). *n*=9 per group in (**d**); *P-value <0.05; **P-value <0.01

According to the cytokine antibody-array results, IGF-1 and LIX were both upregulated 7 DPE. IGF-1 is a cytokine that shows neuroprotective effects and promotes recovery effects in SCI.^12, 13, 14^ Nevertheless, LIX, also known as CXCL5, exhibits potent chemoattractivity for neutrophils in inflammatory microenvironments, which has never been investigated by previous SCI research. To follow the route of epoB exacerbating SCI recovery, we focused on biological effects of elevated LIX after epoB exposure. Figure 5a shows that the MPO assay (*n*=6) illustrated that myeloperoxidase activity obtained from SCI lesions of the SCI-epoB group increased 1.6-fold compared with the SCI-vehicle 10 DPE. Similarly, the infiltrating neutrophils rose in the SCI-epoB group compared with the vehicle control, this effect could be neutralized on different levels by LIX-neutralizing antibody administration 10 DPE (*n*=6) (Figure 5b). BBB scores (*n*=9) show that compared with SCI-epoB, the SCI-epoB-NAM group recovers the best among the three groups from 7 DPE. This score was followed by the SCI-epoB-M-CSF Ab group; it recovers better than SCI-epoB from 21 DPE. At last, SCI-epoB-LIX Ab group presents superior to SCI-epoB at the beginning of 42 DPE (Figure 5d).

## Discussion

Recent data indicate that epoB is a promising strategy for SCI treatment.^1^ On the contrary, in the current study we found that epoB is detrimental for SCI recovery via killing BMDMs, activating microglias, skewing M/M to M1 phenotype in CNS, and recruiting neutrophils to aggravating SCI lesion inflammation in the acute phase, as well as therapeutic benefits for SCI.

These findings reveal that epoB could be a double-edged sword for SCI recovery. These adverse effects are mainly due to epoB reshaping the SCI microenvironment by increasing the cytokine secretion in CSF. Actually, cytokines existing in intercellular washing fluid of lesion site cells modulate the reshaping of the microenvironment.^15^ However, it is very hard to perform the experiments technically by using the intercellular washing fluid of the lesion site. CSF-brain and SC barrier are the weakest in the three parts of blood–brain barriers and the majority of CSF cytokines produced by CNS cells.^16, 17, 18^ As a result, we evaluated the cytokines changing by CSF sample to instead of intercellular washing fluid of lesion site.

Generally speaking, in inflammatory cascade reactions, initially elevated cytokines reach peak within 12–48 h after SCI.^9^ After that, initial cytokines stimulate other cells to secrete downstream cytokines in the second or third cascade reactions in CNS.^19^ However, we found that only M-CSF shows 1.5-fold increase in CSF of SCI-epoB 24 h later, while six kinds of cytokines rose ~3-fold in CSF 72 h after epoB treatment. This phenomenon suggests that the six elevated cytokines may be the second or third cascade reaction after epoB administration. On the other hand, previous research reported that M-CSF could increase expression level of many cytokines, which include TNF-*α*, IL-1, TGF-*β*, and MCP-1.^8, 20^ These results strongly suggest that except for IL-4, M-CSF may be the central and initial cytokine in the SCI superacute stage after epoB administration.

In the CNS, M-CSF modulates the survival, proliferation, maturation, differentiation, recruitment, and activation of M/Ms.^20, 21^ In fact, M-CSF is a milieu-dependent cytokine that exhibits both neuroprotective and/or neurotoxic effects in CNS diseases.^20^ Our study revealed that treating SCI models with epoB generates M-CSF increases in CSF; M/Ms of CNS exposed to both elevated M-CSF and epoB showed contradictory responses. Results showed that the number of CD45^+^ M/Ms in SCI lesion sites decreased as microglias (CD45^−^ M/Ms) of milieu exhibited active states. In other words, the SCI milieu presents cytotoxic effects to homing M/Ms, which are sensitive to microtubule-stabilizing drugs, while resident microglias show activated states, which are sensitive to M-CSF even when they are both exposed to the same microenvironment. How could this dichotomy happen? First of all, one of the most common side effects of epoB is myelosuppression; meanwhile, epoB shows condensed forms in CNS after administration.^1, 22, 23^ Second, recruited M/Ms via ventricle choroid plexus and adjacent SC leptomeninges were still increased because of the elevated M-CSF and MCP-1. Third, microglias are more stable than homing M/Ms in CNS.^24, 25^ It is reasonable to infer that the main reason for this contradiction is that the absorbed epoB exhibit cytotoxicity for homing M/Ms and nonkilling effects for microglias. On the other hand, elevated M-CSF activates microglia rather than dying BMDMs. Then, activated microglia exhibits a variety of neurotoxic effects, including secreting reactive oxygen species, nitric oxide, TNF-*α*, excitatory amino acids, and so on, and also promotes neuroprotection by secreting IGF-1 and increasing macrophage phagocytosis, which is crucial for debris removal.^20, 25^ Unexpectedly, there was no significant change in the M/M number of phagocytic markers of CD68 expression in the SCI-epoB and SCI-vehicle groups. Nevertheless, CD68^+^ M/M shrink and become crenulated morphologically after epoB treatment, which reminds us that epoB shows quite a bit of cytotoxic effects to phagocytic M/Ms and elevated M-CSF stimulated the M/M phagocytosis insufficiently.

Beyond M-CSF, there are five other cytokines elevated in CSF at the same time, which contains three proinflammatory cytokines (IL-1*α*, MCP-1, and TNF-*α*) and two anti-inflammatory cytokines (IL-4 and TGF-*β*).^10, 18, 19, 26^ Many studies reported that M-CSF-treated M/Ms changed to M2 phenotypes.^9, 21, 27^ Interestingly, which phenotypes are lesion site M/Ms tend to when exposing well-balanced anti- and proinflammatory cytokines in CSF? These questions guide us to evaluate the M/M phenotype conversion after epoB treatment.

The M1 phenotype increased in the SCI-epoB lesion site compared with the SCI-vehicle model 3 DPE. It is noteworthy that the M1 phenotype in the SCI-epoB-NAM group is in between; nevertheless, few M2 phenotypes were detected in every group. But over time, phenotype conversion in the M/M substantially weakened in every group 7 DPE. Previous research reported that the M1 phenotype accounted for the overwhelming majority, whereas M2 is a transient and minority polarization, which homes into the lesion site through the second wave after 1 DPI in the acute stage.^9, 25, 28^ It is reasonable to believe that epoB administration induced an M1 phenotype temporary conversion in SCI lesions in the acute SCI injury phase. Moreover, other studies have shown that treatment by both M2-primed cytokine IL-4 and M1-primed cytokines IL-1, MCP-1, and TNF-*α* induced polarization of M/M to M1 phenotype.^26^ Based on our results, both M1 and M2 recruitment increases through SC leptomeninges and choroid plexus. These results strongly suggest that the elevated proinflammatory cytokines IL-1, MCP-1, and TNF-*α* polarized homing monocytes and resident microglias to the M1 phenotype predominantly in the acute SCI injury phase temporarily, while the elevated inflammatory cytokines declined gradually leaving the difference weakened as time goes on. As a matter of fact, only 24 cytokines were evaluated in CSF after epoB treatment, with hundreds of cytokine changes unknown, thus we conducted all six inflammatory cytokines neutralizing *in vivo*. Most noteworthy are the SCI microenvironment polarized M/M into less M1 numbers compared with SCI-epoB, which provides a referable strategy for alleviating the epoB side effects. Locomotion recovery assay agrees with the M/M conversion effects in the SCI milieu again.

Besides IL-1*α* and IL-4, there are two other cytokines, IGF-1 and LIX, that are both elevated 7 DPE. Hamilton and Achuthan^21^ reported that IGF-1 is produced primarily by the activated M/Ms when stimulated by M-CSF in the CNS.^12^ Moreover, LIX was secreted by cells after stimulated by IL-1 or TNF-*α*.^29^ We can infer that the elevated IGF-1 and LIX are the next cascade stage of upregulation of the six cytokines 3 DPE. It has been verified that IGF-1 exhibits both neuroprotective and neurotrophic effects in CNS injury.^30, 31^ We found elevated IGF-1 lasting for more than 7 days after epoB treatment, which may partly explain axon regeneration phenomenon of epoB in SCI. In contrast, LIX is a milieu-dependent cytokine that exhibits the strong attracting effect for neutrophils.^32, 33^ Furthermore, the expression of LIX correlated with the lesion burden and poor prognosis in multiple sclerosis and experimental autoimmune encephalomyelitis.^34^ To our knowledge, little attention has been focused on the role of LIX in SCI. For the above reason, we explored LIX’s effect in SCI. In accordance with the previous report, the level of LIX is proportional to locomotive disability mainly by attracting neutrophils. However, no neutrophil decreasing was observed in the SCI-epoB group, even though neutrophils are the most sensitive hemocytes to epoB. It is mainly due to the time disparity between the attracting neutrophils and epoB treatment.

Like a plethora of SCI research, we only used female rodents as our experimental subjects.^11, 35, 36^ However, its limitation is also obvious, as sex-specific factors bias could lead to inaccurate results. One thing for sure is that estrous cycle affects immunological responses by enhancing or impairing specific responses of immunocompetent cells.^37^ Another sex-specific factor needs to take into account is estrogen. Previous studies reported estrogen is a protective factor by limiting tissue damage and improving functional recovery after rodent SCI.^38^ However researches on human show gender-related differences in recovery after SCI have not been found.^39, 40^ The evidence of estrogen effects in SCI remains inconclusive. Therefore, the finding of our work tends to fit female rodents. Further researches should eliminate the effects due to sex-specific factors in this specific SCI model treated by epoB.

The epoB promotes functional recovery of SCI in many ways; however, current studies show many opposite events, which may counteract the therapy effect. In addition, a few questions are still unclear, such as whether M-CSF is the first inflammatory cascade reactions factor of elevated cytokines? Beyond the elevated cytokines we found, are there any other crucial cytokine changes that influence the prognosis? How do we turn the harmful context into helpful status for functional recovery after epoB administration efficiently? Except for CNS, do any other cells contribute to cytokine sources? Are there any other factors that modulate the protein Tau to facilitate SCI recovery? These findings remind us that the mechanism underlying epoB for SCI treatment is not fully investigated. Further research should focus on the mechanism mentioned above.

## Conclusions

The present study demonstrates that administration of epoB not only has many positive roles in functional recovery but also exhibits many unwanted adverse effects, mainly due to changing the milieu after SCI. Further research should focus on alleviating the side effects.

## Materials and methods

### Animals, SCI surgery, and drug administration

Adult female Sprague–Dawley rats (220±20.0 g, aged 8–10 weeks; Capital Medical University, Beijing, China) were used. All rats were housed in stainless-steel cages (six per cage) in a room kept at 22±1 °C on a 12–12 h light–dark cycle. All rats were acclimatized to their environment for 1 week and free access to food and water. A total of 198 animals were used for all the experiments (Table 1). The study was approved by the Animal Care and Use Committee of Liaoning Medical University in accordance with the Guidelines for the Care and Use of Laboratory Animals published by the US National Institutes of Health.

SCI models were prepared as described previously.^41^ Briefly, the animals were anesthetized with an intraperitoneal injection of chloral hydrate (0.33 ml/kg), and the intact dorsal cord surface was exposed by laminectomy at T9. Subsequently, moderate contusive injury was conducted by impounder striking (2 mm diameter, 12.5 g, 5 cm height) at the surface of T9 SC. The SCI models were maintained on twice-daily bladder evacuation manually until the bladder function of the rat was re-established. After injury, the rats were randomly assigned to various groups. First, in accordance with previous literature, SCI models were treated with epoB (2x0.75 mg/kg; Sellek, Houston, TX, USA) or vehicle (30% PEG400, 5% Ppopylene glycol, 0.5% Tween-80, 0.2% dimethylsulfoxide (DMSO) and 64.3% saline) via intraperitoneal 1  DPI systemically, which is referred to as SCI-epoB or SCI-vehicle. Second, SCI-epoB were randomly administered corresponding neutralizing antibodies according to the experiment design. Rat did not undergo SCI surgery, which was treated with epoB (0.75 mg/kg; Sellek) is referred to as intact-epoB. Rats used for histological analysis were killed by intracardiac perfusion with 0.9% NaCl, followed by 4% paraformaldehyde after anesthesia. Rats used for others assays were killed with an overdose of anesthetic.

### CSF collection

Rats were fixed on a stereotactic instrument after anesthesia by chloral hydrate. The rat’s head was hyperextended at a 135° angle to the body, and the skin around the incision was prepared and sterilized. We then performed a longitudinal incision in the occipital shin and blunt separated the subcutaneous tissue and muscle to expose the foramen magnum region. Next, we inserted the 25 G needle into the cisterna magna and extracted ~100 *μ*l CSF from each rat without blood contamination. Finally, we sutured the skin and muscle, sterilized the incision, and recuperated the rats at 37 °C. CSF was sampled one time a rat for array (*n*=3) and once every other day in experiment groups (animals used for immunofluorescence staining and FC of 3 and 7 DPE) for ELISA (*n*=3) (Table 1). Specifically, we collected CSF sample for ELISA in SCI-vehicle group for immunofluorescence staining of 3 DPE (sample used for ELISA of days 1 and 3); SCI-vehicle group for FC of 3 DPE (day 2); SCI-vehicle group for immunofluorescence staining of 7 DPE (days 4 and 7); SCI-vehicle group for FC of 7 DPE (day 5). SCI-vehicle group of 14 DPE was collected for CSF sample only for ELISA. In the same way, the CSF sample of SCI-epoB group was collected. The M-CSF concentration was measured by ELISA on CSF sample. We analyzed 42 CSF samples without blood contamination for ELISA.

### Cytokine antibody array

RayBiotech Neuro Discovery Array C Series (Raybiotech, Norcross, GA, USA) was used for detecting changes of CSF cytokine levels after epoB or vehicle treatment (*n*=3) and total 30 CSF samples were analyzed. We centrifuged the CSF samples at 1000 × *g* for 5 min after thawing to remove any particulates, aspirated 100 *μ*l CSF samples, and diluted them to 1 ml for all later array procedures. The manufacturer’s instructions were followed for all steps involving the cytokine array. We briefly placed each membrane into a well of the incubation tray and incubated in blocking buffer for 30 min at room temperature. After washing, we added diluted CSF into each well and incubated it overnight at 4 °C. After consecutive washes, we pipetted 1 ml of the prepared detection antibody cocktail into each well and incubated it overnight at 4 °C. With the same washes, 2 ml of HRP-streptavidin was added into each well and incubated overnight at 4 °C. After consecutive washes, we then added 500 *μ*l of the detection buffer mixture onto each membrane and incubated them for 2 min at room temperature. Afterwards, we transferred the membranes to the CCD camera and exposed them. The intensity of the positive control signal was used to normalize the cytokine signal between the two arrays.

### Enzyme-linked immunosorbent assay

The rats’ CSF of the SCI-vehicle and SCI-epoB groups were collected as described above in consecutive time. Level and duration of the increasing M-CSF was measured using ELISA (*n*=3) (Sangon Biotech, Shanghai, China) according to the manufacturer's instructions.

### Administration of neutralizing antibodies

All neutralizing antibodies were intravenously injected into the tail vein every other day after epoB or vehicle administration. The injection doses of neutralizing antibodies are specified according to the published studies, as follows: goat anti-IL-1*α* (200 *μ*g per rat; Peprotech, Rocky Hill, NJ, USA), goat anti-IL-4 (250 *μ*g per rat; R&D, Minneapolis, MN, USA), mouse anti-M-CSF (750 *μ*g per rat; R&D), rabbit anti-MCP-1 (500 *μ*g per rat; Peprotech), rabbit anti-TNF-*α* (100 *μ*g/rat; Peprotech), mouse anti-TGF-*β* (250 *μ*g/rat; Abcam, Cambridge, UK).^9, 42, 43, 44, 45, 46^ A mix of isotype antibody served as the control. The number and start time of neutralizing antibodies injection was based on results of cytokines antibody array. SCI-epoB injected with M-CSF antibody or MCP-1 antibody from 0 DPE until 7 DPE or killing are referred to as SCI-epoB-M-CSF Ab or SCI-epoB-MCP-1 Ab, respectively. SCI-epoB injected with neutralizing antibody mix (IL-1*α*, IL-4, M-CSF, MCP-1, TNF-*α*, and TGF-*β*) from 0 DPE until 7 DPE or killing is referred to as SCI-epoB-NAM, and SCI-epoB injected with LIX (C–X–C motif chemokine 5) antibody from 3 DPE until 14 DPE or killing is referred to as SCI-epoB-LIX Ab.

### Immunofluorescence staining

For *in vivo* experiments, SCI models were anesthetized and killed via intracardiac perfusion with 0.9% NaCl, followed by 4% paraformaldehyde 3, 7 or 10 DPE. Then SC and brain samples were removed and fixed in 4% paraformaldehyde. After 3 days fixation, tissues were equilibrated in paraformaldehyde supplemented with 30% sucrose. After that, the samples were cut into 10-*μ*m sections horizontally, transversely or sagittally, and the slides were kept in a cryoprotective solution at −80 °C.

At the beginning, we warmed the frozen slides to room temperature and washed them once gently in phosphate-buffered solution (PBS), and then we blocked the slides in blocking buffer (PBS supplementary with 1% BSA+5% goat serum). After washing two times gently, the following primary anti-rat antibodies were incubated with the sections in the blocking buffer at 4 °C overnight: APC-conjugated anti-CD11b (1 : 100; Miltenyi, Hamburg Germany), chicken anti-GFAP (1:1000; Abcam), rabbit anti-NeuN (1 : 200; Abcam), rabbit anti-CD45 (1 : 100; Abcam), mouse anti-CD31 (1 : 100; Novus, Littleton, CO, USA), chicken anti-vimentin (1 : 5000; Novus), mouse anti-CD8 (1:100; Abcam), rabbit anti-Iba-1 (1:100; Abcam), rabbit anti-myeloperoxidase (MPO) (1:100; Abcam), mouse anti-CD68 (1 *μ*g/ml; Abcam), mouse anti-M-CSF (1:100; R&D), rabbit anti-M-CSF (1 : 100; Abcam) and mouse anti-O4 (1 : 200; R&D). After overnight incubation, primary antibodies were discarded and slides were rinsed gently in PBS three times. Afterwards, the fluorescent secondary antibodies were added to sections for single or double staining: FITC-conjugated goat anti-rabbit (1 : 500; Bioss, Beijing, China), PE-conjugated goat anti-mouse (1 : 500; Bioss), FITC-conjugated goat anti-mouse (1 : 500; Bioss), PE-conjugated goat anti-rabbit (1 : 500; Bioss), and CY3-conjugated goat anti-chicken (1 : 500; Bioss) for 2 h at room temperature. Finally, nuclei were stained with Prolong Gold Antifade reagent with DAPI (Invitrogen, Carlsbad, CA, USA). Sections were captured with a fluorescent microscope (Olympus, Tokyo, Japan) at equal exposure time.

### Cell culture and viability assays

NR8383 cells (Procell, Wuhan, China) were cultured in DMEM/F12K medium (Gibco-BRL, Grand Island, NY, USA) supplement with 20% heat-inactivated fetal bovine serum (Gibco, Scoresby, VIC, Australia), 50 U/ml penicillin, and 50 *μ*g/ml streptomycin at 37 °C supplied with 5% CO_2_. Cell viability was evaluated by monotetrazolium (MTT) assay and trypan blue staining. In brief, NR8383 cells were planted at a density of 1x10^4^ per well in 96-well plates. After incubation for 24 h, the cells were treated by medium or 2 ng/ml M-CSF, and meanwhile they were also exposed to epoB at the final concentration of 1 or 5 nM. After 3 or 5 days of stimulation, 20 *μ*l MTT (5 mg/ml) was added to each well, and 4 h later, cells were lysed by 100 *μ*l of DMSO. The absorbance was determined at 570 nm on a scanning multiwell spectrophotometer (Tecan, Morrisville, NC, USA). For further assessing the cytotoxic effects of epoB on M/Ms, trypan blue dye technique was used for distinguishing the dead cells. Briefly, NR8383 was plated in 8-well chamber slides (BD-Falcon, Franklin Lakes, NJ, USA) and treated by M-CSF plus epoB as above. Three days after stimulation, slides were centrifuged to sedimentate suspension cells and stained with trypan blue at the final concentration of 0.04% for 3 min at 37 °C. Data were collected as described above.

### Flow cytometry

For assessing the quantitative, phenotypic, polarized, and functional status of M/Ms, the SCI-vehicle, SCI-epoB-M-CSF, SCI-epoB, and SCI-epoB-NAM group (*n*=6) of rats were killed for FC 3 or 7 DPE. For the groups, SCI-epoB-M-CSF group serves as a positive control because only 24 cytokines were detected by our antibody array, and six of these cytokines were elevated in the CSF. Hundreds of cytokines changing were still unknown, so SCI-epoB-NAM was used for detecting the combined effect of all unknown cytokines in the SCI milieu. Sample collection was conducted by dissecting out the pieces of 0.5 cm length SC using the lesion site as a center. We transferred the samples into cold Dulbecco's phosphate-buffered saline (D-PBS) immediately after resection, and then washed the samples two times with cold D-PBS gently to remove blood cells from the surface of the SC. After that, we cut the sample into multiple small segments, and they were digested by trypsin for 30 min at 37 °C. After digestion, trypsin was removed by centrifugation and cells were resuspended in staining buffer (PBS with 2%FBS). Single-cell suspension was obtained from filtering through the 48*μ*m mesh. Samples were stained according to the manufacturer’s instructions in the subsequent steps. Briefly, 1 × 10^6^ isolated cells were stained in 100 *μ*l staining buffer with the following primary antibodies: APC-conjugated anti-CD11b (10 *μ*l/10^6^ cells; Miltenyi), rabbit anti-CD16/32 (2 *μ*g/10^6^ cells; Bioss, Woburn, MA, USA), mouse anti-CD206 (0.2 *μ*g/10^6^ cells; Rosemont, IL, USA), PE-conjugated anti-CD163 (10 *μ*l/10^6^ cells; GeneTex, Irvine, USA), FITC-conjugated anti-CD86 (1 *μ*g/10^6^ cells; BioLegend, San Diego, CA), rabbit anti-TNF-*α* (2 *μ*g/10^6^ cells; Peprotech, Rocky Hill, NJ, USA), mouse anti-TGF-*β* (2.5 *μ*g/10^6^ cells; Abcam, Cambridge, UK), mouse anti-CD68 (1 *μ*g/10^6^ cells; Abcam, Cambridge, MA, USA), and rabbit anti-CD45(1 *μ*g/10^6^ cells; Abcam, Cambridge, MA, USA). After incubation for 30 min at 4 °C with the first antibodies, and washing twice by staining buffer, the following fluorescent-labeled secondary antibodies were added to bind corresponding primary non-fluorescent antibodies: FITC-conjugated goat anti-rabbit (0.5 *μ*l/10^6^ cells; Bioss, China) and PE-conjugated goat anti-mouse (0.5 *μ*l/10^6^ cells; Bioss, China). After incubating and washing the same as the last step, cells were fixed with 1% paraformaldehyde before analysis by FC. A total of 50 000 events were collected and analyzed on FACSCalibur Flow Cytometer (BD Biosciences, San Jose, CA, USA) using the FlowJo Software (Tree Star, Inc., Ashland, OR, USA). Isotype-matched antibody-stained cells, positive-stained cells, and unstained cells were used as controls to gate the cell populations of interest.

To detect the elevated M-CSF’s proliferative effect on M/Ms, the NR8383 cell line and CFSE cell proliferation assay were used *in vitro*. NR8383 cells were stained following the manufacturer’s instructions (Beyotime, Hangzhou, Zhejiang, China). In brief, cultured NR8383 was digested and resuspended in CFSE staining buffer without FBS as 2 × 10^6^ cells/ml for 10 min at 37 °C. After incubation, adding FBS, washing, and labeling, cells were seeded in six-well plates as 5 × 10^5^ cells/well. Subsequently, the cells were treated with medium or 2 ng/ml rat recombinant M-CSF while being exposed to 1 nM/5 nM epoB or not. Either 3 or 5 days after labeling, cells were analyzed on FACSCalibur flow cytometer as described above.

### M/M labeling *in vivo*

For assessing the cytotoxic effect of epoB in SCI milieu M/Ms, fluorophore-conjugated primary antibody was used for labeling the M/Ms as described previously.^47^ In short, 1 ml saline containing 50 *μ*l APC-conjugated anti-CD11b (Miltenyi) was injected via the coccygeal vein in SCI model 1 DPE. SC tissues were taken for visualization of recruited M/Ms with immunofluorescence staining (*n*=3) 3 DPE.

### MPO assay

MPO activity in the SC of the SCI-vehicle, SCI-epoB, and SCI-epoB-LIX Ab group 10 DPE were determined by MPO Detection Kit (*n*=6) (Jiancheng Bioengineering Institute, Nanjing, China). The experiment was conducted according to the instructions. Briefly, SC tissue was washed by cold PBS to remove blood cells from the surface. Then tissues were homogenized in 0.5% hexadecyltrimethylammonium hydroxide PBS solution and centrifuged. After centrifugation, the supernatant was transferred into PBS (pH 6.0) containing 0.17 mg/ml 3,3′-dimethoxybenzidine and 0.0005% H_2_O_2_. Afterwards, supernatant MPO catalyzed H_2_O_2_-dependent oxidation of 3,3′-dimethoxybenzidine to produce yellow compound, which could be measured by 460 nm.

### Quantification of immunostaining

Quantification of immunolabeling was estimated by unbiased observers after removing the background threshold level using the Image-Pro Plus Software 5.1 (Media Cybernetics Inc., Atlanta, GA, USA) as described previously.^12^ Every group contained six or three (M/M labeling *in vivo*) animals, and the SCs from rats were cut into five 10-*μ*m-thick sections spaced 20 *μ*m apart serially through the entire injury site. The target area was a range of ±1000 *μ*m surrounding the lesion epicenter, and the counting frame size was 550 *μ*m × 550 *μ*m. The injury size was detected by Luxol/Nissel staining, and the lesion epicenter was identified as the unlabeled region by Luxol. Quantifications of the M/M and neutrophils numbers, density per counting frame, and density per cell were performed using specific tools of the Image-Pro Plus Software (Media Cybernetics, Inc., Silver Spring, MD, USA).

### Assessment of locomotion recovery

Locomotion recovery was evaluated by the Basso, Beattie, and Bresnahan (BBB) open-field locomotor rating scale (*n*=9) as described by Basso *et al.*^48^ Briefly, two investigators, who were not aware of the experiment design, operated the behavioral tests. BBB scores were observed and recorded at 1, 3, 5, and 7 DPE and then weekly until 7 weeks.

### Statistical analysis

Data were analyzed using SPSS Software, version 11.0 (Chicago, IL, USA) and expressed as the mean±S.D. The differences among groups were compared and analyzed by one-way ANOVA, followed by the Fisher's LSD procedure. The rate of the group was analyzed by *χ*^2^ test or Fisher's exact test. Heteroscedastic data was performed with the Mann–Whitney *U*-test to evaluate differences among the experimental groups. *P*<0.05 was considered statistically significant.

## Figures and Tables

**Table 1:** Number of animals used for all experimental groups

**Group/time of killing**	**1 DPE**	**3 DPE**	**7 DPE**	**10 DPE**	**14 DPE**	**49 DPE**
Intact-epoB		3 (Array)				
SCI-vehicle (days post vehicle treatment)	3 (Array)	3 (Array) 6 (Immunofluorescence staining) contain ELISA (*n*=3) 6 (FC) contain ELISA (*n*=3) 3 (M/M labeling *in vivo*)	3 (Array) 6 (immunofluorescence staining) contain ELISA (*n*=3) 6 (FC) contain ELISA (*n*=3)	6 (MPO assay) 6 (immunofluorescence staining)	3 (Array) 3 (ELISA)	
SCI-epoB	3 (Array)	6 (Array) 6 (immunofluorescence staining) contain ELISA (*n*=3) 6 (FC) contain ELISA (*n*=3) 3 (M/M labeling *in vivo*)	3 (Array) 6 (immunofluorescence staining) contain ELISA (*n*=3) 6 (FC) contain ELISA (*n*=3)	6 (MPO assay) 6 (immunofluorescence staining)	3 (Array) 3 (ELISA)	9 (BBB)
SCI-epoB-M-CSF Ab		6 (immunofluorescence staining) 6 (FC)	6 (FC)			9 (BBB)
SCI-epoB-MCP-1 Ab		6 (immunofluorescence staining)				
SCI-epoB-NAM		6 (FC)	6 (FC)			9 (BBB)
SCI-epoB -LIX Ab				6 (MPO assay) 6 (immunofluorescence staining)		9 (BBB)
						
Total number=198	6	66	42	36	12	36

Abbreviations: Ab, antibody; BBB, Basso, Beattie, and Bresnahan; DPE, day post epoB treatment; ELISA, enzyme-linked immunosorbent assay; epoB, epothilone B; FC, flow cytometry; LIX, C–X–C motif chemokine 5; MCP-1, monocyte chemoattractant protein-1; M-CSF, macrophage colony-stimulating factor; M/M, microglia/macrophages/monocyte; MPO, myeloperoxidase; NAM, neutralizing antibody mix; SCI, spinal cord injury
